# Supplementary material for: Vibrational and electronic properties of Np[image]O[image] from experimental spectroscopy and first principles calculations
Source: Sci Rep. 2026 Mar 31;16:10883. doi: 10.1038/s41598-026-36720-x (PMC13039402; doi:10.1038/s41598-026-36720-x)
Supplement: Supplementary file 1 — Supplementary Information. [file 41598_2026_36720_MOESM1_ESM.pdf]

Electronic and Lattice Dynamics of Np_2O_5

Binod K Rai[1*], Shuxiang Zhou[2], Benjamin R. Heiner[3], Gia Thinh Tran[1],
Jennifer E. S. Szymanowski[4], Santosh KC[5], Thomas Shehee[1],
Peter C. Burns[4] Miles F. Beaux II[3], Luke R Sadergaski[6]

[1]Savannah River National Laboratory, Aiken, SC 29808, USA.

[2]Idaho National Laboratory, Idaho Falls, ID 83415, USA.

[3]Los Alamos National Laboratory, P.O. Box 1663, Los Alamos, NM 87545, USA.

[4]Department of Civil and Environmental Engineering and Earth Sciences, University of Notre Dame, Notre Dame, IN 46556 USA.

[5]Mechanical Engineering, San Diego State University, San Diego, CA 92182 USA.

[6]Oak Ridge National Laboratory, Oak Ridge, TN 37830, USA.

*Corresponding author. Emails:binod.rai@srnl.doe.gov

Supplementary Information

Here, we report experimental Raman spectra, and computed results of lattice structure, Raman peaks, dielectric constants, Born effective charge, Infrared (IR) spectra, VASP inputs, linear response ansatz for DFT+U, and all vibrational modes.

1 Experimental Raman Spectra

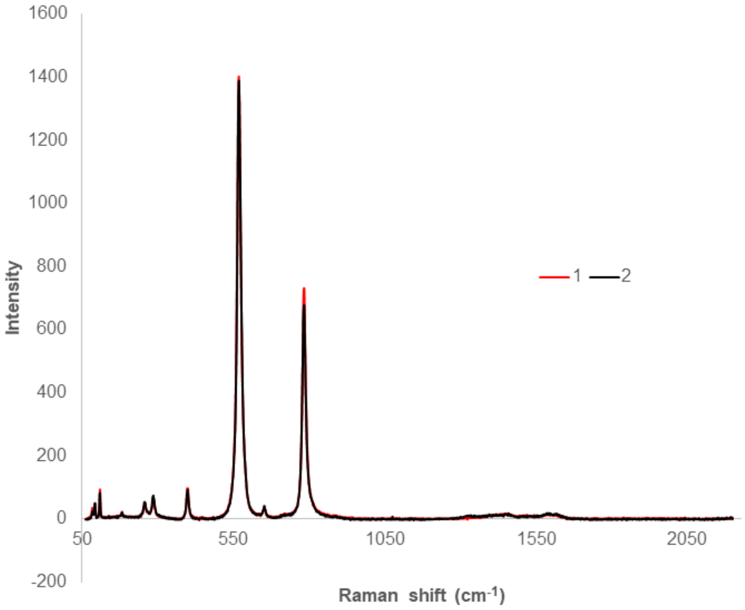

Figure 1: Experimental Raman spectra of two single crystals of Np_2O_5, showing reproducible and well-defined peaks.

2 Computed Lattice Structure

The computed lattice parameters and cell volume are tabulated in Table 1, compared to our XRD measurements and previous experimental and computational studies. (*1, 2*) Generally, good agreements are obtained for the lattice parameters, which are slightly over-predicted by GGA+U and slightly under-predicted by LDA+U. Overall, LDA+U produces slightly better agreement with experiment than GGA+U, *i.e.* the error on volume prediction is -2.1% from LDA+U compared to 4.2% from GGA+U; therefore, LDA+U is applied in further calculations. The details of computed lattice structures are provided in Table 2.

Table 1: The lattice parameters and cell volume measured from DFT+U calculations and experiments in this work, compared with literature values (*1, 2*).

	This work			Literature	
	XRD	LDA	GGA	Expt. (*1*)	Calc. (*2*)
a(Å)	8.168	8.094	8.283	8.168	8.28
b(Å)	6.584	6.555	6.675	6.584	6.69
c(Å)	9.313	9.181	9.424	9.313	9.46
β (°)	116.09	115.34	115.96	116.09	119.20
V(Å^3)	449.8	440.3	468.5	449.8	457.4

Table 2: Computed lattice structure of Np_2O_5 by using LDA+U+SOC and GGA+U+SOC (U = 3 eV).

	LDA	GGA
	Lattice Vectors (Å)	
**a**	(5.6285, 0.0342, 0.3628)	(5.7739, -0.0086, 0.0073)
**b**	(-1.8445, 5.3187, -0.3626)	(-1.9078, 5.4498, -0.0136)
**c**	(-3.2810, -1.6939, 7.2035)	(-2.8269, -2.0756, 7.4483)
	Atomic Coordinates	
Np1	(0.2909, 0.5409, 0.2500)	(0.2907, 0.5408, 0.2499)
Np2	(0.5000, 1.0000, 0.0000)	(0.5000, 0.0000, 0.0000)
Np3	(0.9999, 0.9999, 0.5000)	(0.0000, 1.0000, 0.5000)
Np4	(0.7091, 0.4589, 0.7500)	(0.7093, 0.4592, 0.7501)
O1	(0.3512, 0.1007, 0.7499)	(0.3500, 0.0993, 0.7499)
O2	(0.8336, 0.2377, 0.6442)	(0.8308, 0.2420, 0.6429)
O3	(0.1665, 0.7625, 0.3558)	(0.1692, 0.7580, 0.3571)
O4	(0.4077, 0.3117, 0.1440)	(0.4021, 0.3135, 0.1427)
O5	(0.6488, 0.8992, 0.2501)	(0.6500, 0.9006, 0.2501)
O6	(0.5925, 0.6885, 0.8561)	(0.5979, 0.6865, 0.8573)
O7	(0.8418, 0.2871, 0.0321)	(0.8382, 0.2840, 0.0331)
O8	(0.7447, 0.6909, 0.5324)	(0.7489, 0.6958, 0.5336)
O9	(0.1582, 0.7128, 0.9679)	(0.1618, 0.7160, 0.9668)
O10	(0.2553, 0.3092, 0.4676)	(0.2512, 0.3043, 0.4665)

3 Computed Raman Peaks

Table 3: Computed Raman peaks of Np_2O_5 by using LDA+U+SOC (U = 3 eV). The Raman peaks that are noticeable in our experiments are marked by bold text. The peak mode is assigned corresponding to the $C2h$ point group.

Wavenumber (cm^{-1})	Mode	Intensity (Å^4 amu^{-1})	Linewidth (cm^{-1})
**88.39**	B_g	10.68	1.46
**100.37**	B_g	10.27	1.36
**114.61**	A_g	14.36	0.89
132.46	B_g	0.27	1.86
**176.99**	A_g	11.60	2.71
187.23	B_g	7.19	1.82
209.48	A_g	2.25	2.13
**267.85**	A_g	50.27	4.40
269.09	B_g	29.34	2.61
275.62	A_g	13.47	2.34
291.27	B_g	41.70	2.05
**293.20**	B_g	266.65	5.04
**396.97**	A_g	429.07	4.12
444.24	B_g	2.29	5.09
**522.39**	A_g	8113.48	7.49
**662.09**	B_g	305.24	5.89
**750.02**	A_g	6711.11	8.07
825.80	B_g	67.97	5.15

4 Computed Dielectric Constants and Born Effective Charges

Dielectric constants matrix:

$$\epsilon = \begin{pmatrix} 4.78 & 0.12 & -0.12 \\ 0.12 & 4.87 & 0.08 \\ -0.13 & 0.09 & 4.44 \end{pmatrix}$$

Born effective charges for each atom:

$$\text{Np1, Np4:} \quad Z^* = \begin{pmatrix} 5.77 & -0.52 & -0.77 \\ -0.55 & 5.39 & 0.56 \\ -0.85 & 0.59 & 4.51 \end{pmatrix}$$

$$\text{Np2:} \quad Z^* = \begin{pmatrix} 5.24 & 0.17 & -0.28 \\ 0.63 & 6.46 & -0.51 \\ -0.23 & -0.53 & 5.49 \end{pmatrix}$$

$$\text{Np3:} \quad Z^* = \begin{pmatrix} 6.08 & 0.91 & 0.37 \\ 0.47 & 5.62 & 0.43 \\ 0.43 & 0.39 & 5.48 \end{pmatrix}$$

$$\text{O1, O5:} \quad Z^* = \begin{pmatrix} -1.75 & -0.29 & -1.19 \\ -0.29 & -1.96 & 0.84 \\ -1.18 & 0.84 & -3.89 \end{pmatrix}$$

$$\text{O2, O3:} \quad Z^* = \begin{pmatrix} -3.05 & 1.49 & 1.38 \\ 1.64 & -1.64 & -0.92 \\ 1.41 & -0.90 & -1.43 \end{pmatrix}$$

$$\text{O4, O6:} \quad Z^* = \begin{pmatrix} -2.77 & 1.74 & 1.33 \\ 1.58 & -1.92 & -1.00 \\ 1.31 & -1.03 & -1.43 \end{pmatrix}$$

$$\text{O7, O9:} \quad Z^* = \begin{pmatrix} -2.22 & -1.59 & -0.72 \\ -1.56 & -2.67 & -0.18 \\ -0.75 & -0.22 & -1.62 \end{pmatrix}$$

$$\text{O8, O10:} \quad Z^* = \begin{pmatrix} -1.64 & -1.36 & -0.06 \\ -1.38 & -3.25 & 0.74 \\ -0.03 & 0.79 & -1.63 \end{pmatrix}$$

5 Infrared Spectra

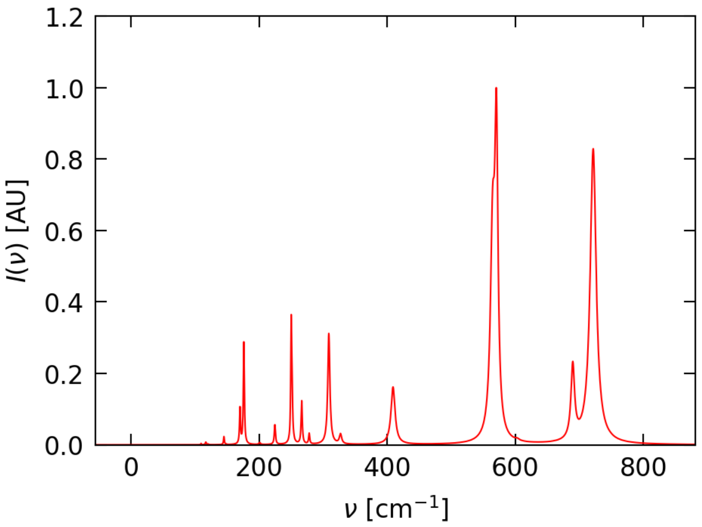

Figure 2: Computed infrared spectra of Np_2O_5 using LDA+U+SOC (U = 3 eV).

6 VASP Inputs

VASP INCAR file:

```
EDIFF = 1e-7
EDIFFG = 1e-3
ENCUT = 520
ISIF = 3
ISMEAR = -5
ISPIN = 2
LASPH = True
LMAXMIX = 6
LORBIT = 11
LREAL = .False.
ADDGRID=.TRUE.
NELM = 100
NSW = 99
PREC = Accurate
SIGMA = 0.05
ISYM = -1
LSORBIT = .TRUE.
LORBMOM=.TRUE.
LDAU = .T.
LDATYPE = 2
LDAUL = 3  -1
LDAUU = 3.0  0.0
LDAUJ = 0.0  0.0
LDAUPRINT = 2
LMAXMIX = 6
```

VASP POTCAR: PAW Np 17Apr2000 and PAW O 22Mar2012

Please refer SI Section 1.2 for POSCAR, and the main text for KPOINTS settings.

7 Linear Response Ansatz for DFT+U

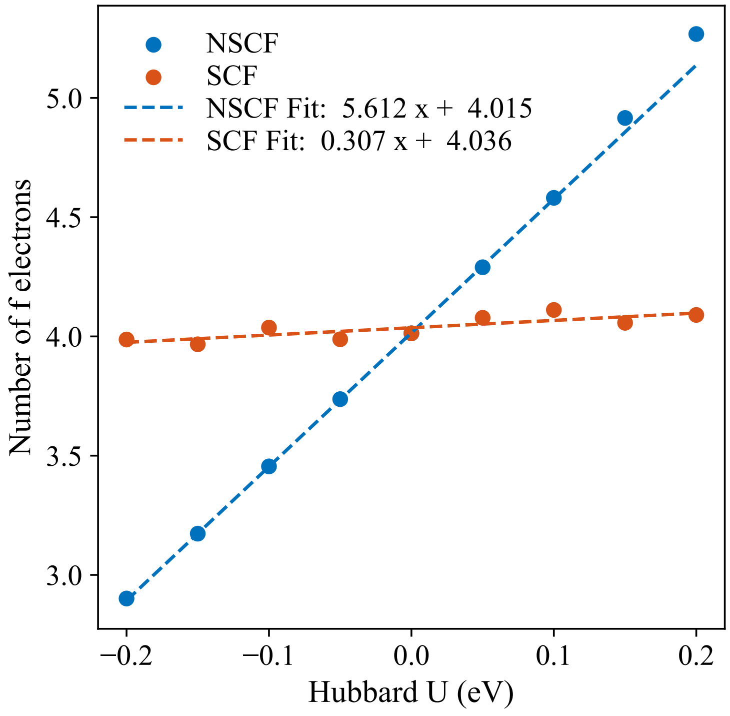

Figure 3: Calculation results of the U parameter for the DFT+U treatment of Np f-electrons in Np_2O_5 using the linear response ansatz. The dots represent calculation results, while the dashed lines are linear fitting; the computed U = 3.1 eV.

8 Vibrational Modes

Here we provide the computed vibrational modes for each phonon mode, in the format of a v_sim ascii file:

```
# Phonopy generated file for v_sim 3.6
    5.640330570   -1.831719056    5.335454072
   -2.821128575   -2.012336493    7.315469589
   -0.055438108    2.383007921    1.828662585 Np
    0.988219417    5.335345830    0.000074277 Np
    2.397923164    4.329062717    3.657710148 Np
    1.043023015    0.939226374    5.486524693 Np
   -0.319425634   -0.971671045    5.485845731  O
    2.449172632   -0.028157311    4.712317963  O
   -1.461492081    3.352474346    2.602887170  O
    1.322236066    1.373094315    1.053473144  O
    1.306577458    4.294249568    1.829605595  O
   -0.334158517    1.950791674    6.262477911  O
    4.131708698    1.467036367    0.234886524  O
    1.432581193    2.614843944    3.894825953  O
   -3.143974939    1.855694737    7.080326457  O
   -0.445561282    0.708445932    3.420934175  O
#metaData: qpt=[0.000000;0.000000;0.000000;-0.036352 \
#; 0.005269; -0.010722; -0.027468; 0.000000; 0.000000; 0.000000 \
#; 0.005355; -0.010917; -0.027619; 0.000000; 0.000000; 0.000000 \
#; 0.005348; -0.010833; -0.027573; 0.000000; 0.000000; 0.000000 \
#; 0.005285; -0.010719; -0.027473; 0.000000; 0.000000; 0.000000 \
#; 0.005772; -0.011235; -0.027843; 0.000000; 0.000000; 0.000000 \
#; 0.005537; -0.010445; -0.027286; 0.000000; 0.000000; 0.000000 \
#; 0.005527; -0.010448; -0.027269; 0.000000; 0.000000; 0.000000 \
#; 0.005231; -0.011190; -0.027077; 0.000000; 0.000000; 0.000000 \
```

```
#; 0.005769; -0.011242; -0.027847; 0.000000; 0.000000; 0.000000 \
#; 0.005268; -0.011156; -0.027096; 0.000000; 0.000000; 0.000000 \
#; 0.005318; -0.010882; -0.027578; 0.000000; 0.000000; 0.000000 \
#; 0.005139; -0.010701; -0.027347; 0.000000; 0.000000; 0.000000 \
#; 0.005330; -0.010900; -0.027587; 0.000000; 0.000000; 0.000000 \
#; 0.005132; -0.010698; -0.027343; 0.000000; 0.000000; 0.000000 \
# ]
#metaData: qpt=[0.000000;0.000000;0.000000;-0.026628 \
#; 0.026937; -0.009629; 0.008997; 0.000000; 0.000000; 0.000000 \
#; 0.027044; -0.009659; 0.009041; 0.000000; 0.000000; 0.000000 \
#; 0.027076; -0.009671; 0.009040; 0.000000; 0.000000; 0.000000 \
#; 0.026940; -0.009629; 0.008986; 0.000000; 0.000000; 0.000000 \
#; 0.027336; -0.009792; 0.008963; 0.000000; 0.000000; 0.000000 \
#; 0.027091; -0.009453; 0.008891; 0.000000; 0.000000; 0.000000 \
#; 0.027072; -0.009465; 0.008899; 0.000000; 0.000000; 0.000000 \
#; 0.026720; -0.009739; 0.008786; 0.000000; 0.000000; 0.000000 \
#; 0.027318; -0.009779; 0.008977; 0.000000; 0.000000; 0.000000 \
#; 0.026719; -0.009755; 0.008790; 0.000000; 0.000000; 0.000000 \
#; 0.026725; -0.009361; 0.009174; 0.000000; 0.000000; 0.000000 \
#; 0.026811; -0.009536; 0.009071; 0.000000; 0.000000; 0.000000 \
#; 0.026732; -0.009362; 0.009174; 0.000000; 0.000000; 0.000000 \
#; 0.026808; -0.009530; 0.009074; 0.000000; 0.000000; 0.000000 \
# ]
#metaData: qpt=[0.000000;0.000000;0.000000;-0.020567 \
#; 0.012047; 0.026306; -0.007990; 0.000000; 0.000000; 0.000000 \
#; 0.012063; 0.026335; -0.008084; 0.000000; 0.000000; 0.000000 \
#; 0.012083; 0.026339; -0.007974; 0.000000; 0.000000; 0.000000 \
#; 0.012053; 0.026297; -0.007988; 0.000000; 0.000000; 0.000000 \
#; 0.012211; 0.026498; -0.008053; 0.000000; 0.000000; 0.000000 \
#; 0.012170; 0.026183; -0.007836; 0.000000; 0.000000; 0.000000 \
```

```
#; 0.012189; 0.026237; -0.007846; 0.000000; 0.000000; 0.000000 \
#; 0.011946; 0.026302; -0.007944; 0.000000; 0.000000; 0.000000 \
#; 0.012218; 0.026494; -0.008053; 0.000000; 0.000000; 0.000000 \
#; 0.011967; 0.026308; -0.007936; 0.000000; 0.000000; 0.000000 \
#; 0.012036; 0.026363; -0.008015; 0.000000; 0.000000; 0.000000 \
#; 0.012243; 0.026258; -0.008027; 0.000000; 0.000000; 0.000000 \
#; 0.012044; 0.026356; -0.008019; 0.000000; 0.000000; 0.000000 \
#; 0.012209; 0.026282; -0.008016; 0.000000; 0.000000; 0.000000 \
# ]
#metaData: qpt=[0.000000;0.000000;0.000000;2.649970 \
#; -0.008260; 0.005673; -0.023708; 0.000000; 0.000000; 0.000000 \
#; -0.000033; 0.000038; 0.000192; 0.000000; 0.000000; 0.000000 \
#; 0.000016; -0.000048; 0.000168; 0.000000; 0.000000; 0.000000 \
#; 0.008245; -0.005680; 0.023307; 0.000000; 0.000000; 0.000000 \
#; 0.047289; -0.033788; -0.016581; 0.000000; 0.000000; 0.000000 \
#; 0.053201; 0.054748; 0.031243; 0.000000; 0.000000; 0.000000 \
#; -0.052822; -0.054534; -0.031216; 0.000000; 0.000000; 0.000000 \
#; 0.034067; 0.066642; -0.029864; 0.000000; 0.000000; 0.000000 \
#; -0.047399; 0.033863; 0.017046; 0.000000; 0.000000; 0.000000 \
#; -0.034015; -0.066397; 0.029384; 0.000000; 0.000000; 0.000000 \
#; 0.036556; -0.020809; -0.023935; 0.000000; 0.000000; 0.000000 \
#; -0.031498; 0.027765; 0.023634; 0.000000; 0.000000; 0.000000 \
#; -0.036536; 0.020778; 0.024159; 0.000000; 0.000000; 0.000000 \
#; 0.031728; -0.027941; -0.023518; 0.000000; 0.000000; 0.000000 \
# ]
#metaData: qpt=[0.000000;0.000000;0.000000;3.008706 \
#; -0.017488; 0.013111; -0.023704; 0.000000; 0.000000; 0.000000 \
#; 0.000060; -0.000005; -0.000132; 0.000000; 0.000000; 0.000000 \
#; 0.000020; -0.000055; -0.000125; 0.000000; 0.000000; 0.000000 \
#; 0.017468; -0.013081; 0.023988; 0.000000; 0.000000; 0.000000 \
```

```
#; 0.002575; -0.001823; 0.010388; 0.000000; 0.000000; 0.000000 \
#; -0.016623; -0.063194; 0.027336; 0.000000; 0.000000; 0.000000 \
#; 0.015919; 0.062204; -0.026983; 0.000000; 0.000000; 0.000000 \
#; -0.053008; -0.033858; -0.028115; 0.000000; 0.000000; 0.000000 \
#; -0.003376; 0.002412; -0.010179; 0.000000; 0.000000; 0.000000 \
#; 0.053303; 0.034569; 0.028102; 0.000000; 0.000000; 0.000000 \
#; -0.036050; 0.038116; 0.013376; 0.000000; 0.000000; 0.000000 \
#; 0.048222; -0.022110; -0.013501; 0.000000; 0.000000; 0.000000 \
#; 0.036225; -0.038123; -0.013669; 0.000000; 0.000000; 0.000000 \
#; -0.048101; 0.021869; 0.013123; 0.000000; 0.000000; 0.000000 \
# ]
#metaData: qpt=[0.000000;0.000000;0.000000;3.271557 \
#; 0.007568; -0.005383; 0.018107; 0.000000; 0.000000; 0.000000 \
#; -0.025424; -0.015320; -0.020162; 0.000000; 0.000000; 0.000000 \
#; 0.007056; 0.028149; -0.020211; 0.000000; 0.000000; 0.000000 \
#; 0.007747; -0.005385; 0.018202; 0.000000; 0.000000; 0.000000 \
#; 0.014726; -0.010243; -0.025400; 0.000000; 0.000000; 0.000000 \
#; 0.028475; 0.023217; 0.019143; 0.000000; 0.000000; 0.000000 \
#; 0.027742; 0.022593; 0.018774; 0.000000; 0.000000; 0.000000 \
#; -0.013740; -0.032067; 0.014629; 0.000000; 0.000000; 0.000000 \
#; 0.014203; -0.009924; -0.025197; 0.000000; 0.000000; 0.000000 \
#; -0.013821; -0.032449; 0.014822; 0.000000; 0.000000; 0.000000 \
#; -0.022006; -0.021928; 0.013061; 0.000000; 0.000000; 0.000000 \
#; 0.015277; 0.026350; 0.011682; 0.000000; 0.000000; 0.000000 \
#; -0.022137; -0.021814; 0.013135; 0.000000; 0.000000; 0.000000 \
#; 0.015582; 0.026157; 0.011492; 0.000000; 0.000000; 0.000000 \
# ]
#metaData: qpt=[0.000000;0.000000;0.000000;3.435749 \
#; 0.024704; 0.033607; -0.000240; 0.000000; 0.000000; 0.000000 \
#; -0.000227; -0.000014; 0.000047; 0.000000; 0.000000; 0.000000 \
```

```
#; 0.000212; -0.000025; -0.000002; 0.000000; 0.000000; 0.000000 \
#; -0.024698; -0.033573; 0.000167; 0.000000; 0.000000; 0.000000 \
#; -0.009249; -0.012601; 0.000093; 0.000000; 0.000000; 0.000000 \
#; -0.029405; -0.037552; -0.001490; 0.000000; 0.000000; 0.000000 \
#; 0.029862; 0.037247; 0.002482; 0.000000; 0.000000; 0.000000 \
#; 0.027955; 0.041026; -0.001826; 0.000000; 0.000000; 0.000000 \
#; 0.009390; 0.012619; -0.000079; 0.000000; 0.000000; 0.000000 \
#; -0.028469; -0.040754; 0.000691; 0.000000; 0.000000; 0.000000 \
#; 0.011584; -0.009689; -0.006297; 0.000000; 0.000000; 0.000000 \
#; 0.012056; -0.007292; -0.005925; 0.000000; 0.000000; 0.000000 \
#; -0.012391; 0.009856; 0.006874; 0.000000; 0.000000; 0.000000 \
#; -0.011164; 0.006927; 0.005611; 0.000000; 0.000000; 0.000000 \
# ]
#metaData: qpt=[0.000000;0.000000;0.000000;3.493099 \
#; 0.008499; -0.006125; 0.020089; 0.000000; 0.000000; 0.000000 \
#; 0.010059; 0.027513; -0.019795; 0.000000; 0.000000; 0.000000 \
#; -0.025016; -0.016366; -0.018960; 0.000000; 0.000000; 0.000000 \
#; 0.008055; -0.006093; 0.020309; 0.000000; 0.000000; 0.000000 \
#; -0.014917; 0.010504; -0.011608; 0.000000; 0.000000; 0.000000 \
#; -0.004644; -0.021695; 0.017277; 0.000000; 0.000000; 0.000000 \
#; -0.004690; -0.022349; 0.017023; 0.000000; 0.000000; 0.000000 \
#; 0.021561; 0.009809; 0.022266; 0.000000; 0.000000; 0.000000 \
#; -0.014871; 0.010565; -0.011581; 0.000000; 0.000000; 0.000000 \
#; 0.020498; 0.008997; 0.022375; 0.000000; 0.000000; 0.000000 \
#; 0.013991; 0.028125; -0.021297; 0.000000; 0.000000; 0.000000 \
#; -0.025641; -0.019223; -0.018377; 0.000000; 0.000000; 0.000000 \
#; 0.013314; 0.028664; -0.020932; 0.000000; 0.000000; 0.000000 \
#; -0.025159; -0.019547; -0.018618; 0.000000; 0.000000; 0.000000 \
# ]
#metaData: qpt=[0.000000;0.000000;0.000000;3.605004 \
```

```
#; 0.001918; 0.002259; 0.000529; 0.000000; 0.000000; 0.000000 \
#; -0.031444; 0.020205; 0.011822; 0.000000; 0.000000; 0.000000 \
#; 0.026650; -0.026040; -0.012681; 0.000000; 0.000000; 0.000000 \
#; 0.002361; 0.002685; 0.000242; 0.000000; 0.000000; 0.000000 \
#; 0.000373; 0.001731; -0.000293; 0.000000; 0.000000; 0.000000 \
#; 0.022298; -0.008718; 0.039152; 0.000000; 0.000000; 0.000000 \
#; 0.021865; -0.008538; 0.038648; 0.000000; 0.000000; 0.000000 \
#; -0.013090; 0.019685; -0.037242; 0.000000; 0.000000; 0.000000 \
#; 0.000084; 0.001702; -0.000113; 0.000000; 0.000000; 0.000000 \
#; -0.012880; 0.019939; -0.037633; 0.000000; 0.000000; 0.000000 \
#; -0.033284; 0.020738; 0.022920; 0.000000; 0.000000; 0.000000 \
#; 0.027455; -0.027783; -0.024940; 0.000000; 0.000000; 0.000000 \
#; -0.033289; 0.020900; 0.022706; 0.000000; 0.000000; 0.000000 \
#; 0.027181; -0.027525; -0.024834; 0.000000; 0.000000; 0.000000 \
# ]
#metaData: qpt=[0.000000;0.000000;0.000000;3.971398 \
#; -0.023169; 0.017271; 0.027431; 0.000000; 0.000000; 0.000000 \
#; 0.000372; 0.000174; -0.000103; 0.000000; 0.000000; 0.000000 \
#; -0.000314; -0.000213; 0.000096; 0.000000; 0.000000; 0.000000 \
#; 0.023070; -0.017229; -0.027306; 0.000000; 0.000000; 0.000000 \
#; 0.050846; -0.036698; -0.030821; 0.000000; 0.000000; 0.000000 \
#; 0.035734; -0.010401; -0.006731; 0.000000; 0.000000; 0.000000 \
#; -0.034291; 0.012452; 0.006982; 0.000000; 0.000000; 0.000000 \
#; -0.022515; 0.028722; 0.007531; 0.000000; 0.000000; 0.000000 \
#; -0.048802; 0.035312; 0.029658; 0.000000; 0.000000; 0.000000 \
#; 0.020976; -0.030584; -0.007679; 0.000000; 0.000000; 0.000000 \
#; -0.007531; -0.002618; 0.009676; 0.000000; 0.000000; 0.000000 \
#; -0.001583; -0.007964; -0.009480; 0.000000; 0.000000; 0.000000 \
#; 0.007231; 0.003931; -0.010003; 0.000000; 0.000000; 0.000000 \
#; 0.000086; 0.007952; 0.009382; 0.000000; 0.000000; 0.000000 \
```

```
# ]
#metaData: qpt=[0.000000;0.000000;0.000000;4.336895 \
#; -0.004513; 0.003742; -0.001123; 0.000000; 0.000000; 0.000000 \
#; 0.012471; 0.006448; -0.000045; 0.000000; 0.000000; 0.000000 \
#; -0.005958; -0.012374; 0.005490; 0.000000; 0.000000; 0.000000 \
#; -0.005090; 0.004055; -0.000082; 0.000000; 0.000000; 0.000000 \
#; 0.076586; -0.052985; -0.041335; 0.000000; 0.000000; 0.000000 \
#; 0.050019; 0.065904; 0.015900; 0.000000; 0.000000; 0.000000 \
#; 0.050968; 0.065783; 0.015299; 0.000000; 0.000000; 0.000000 \
#; -0.053024; -0.063163; -0.006661; 0.000000; 0.000000; 0.000000 \
#; 0.078437; -0.054259; -0.042572; 0.000000; 0.000000; 0.000000 \
#; -0.053024; -0.061637; -0.006322; 0.000000; 0.000000; 0.000000 \
#; -0.020517; 0.037025; 0.003663; 0.000000; 0.000000; 0.000000 \
#; -0.035570; 0.002124; 0.001141; 0.000000; 0.000000; 0.000000 \
#; -0.020487; 0.036765; 0.003825; 0.000000; 0.000000; 0.000000 \
#; -0.035727; 0.001966; 0.000915; 0.000000; 0.000000; 0.000000 \
# ]
#metaData: qpt=[0.000000;0.000000;0.000000;4.437434 \
#; -0.002496; -0.001818; 0.000105; 0.000000; 0.000000; 0.000000 \
#; -0.008544; 0.011897; 0.021601; 0.000000; 0.000000; 0.000000 \
#; 0.013805; -0.006606; -0.021233; 0.000000; 0.000000; 0.000000 \
#; -0.002483; -0.002188; 0.000208; 0.000000; 0.000000; 0.000000 \
#; 0.003801; -0.017298; -0.005899; 0.000000; 0.000000; 0.000000 \
#; -0.027517; 0.031285; -0.086248; 0.000000; 0.000000; 0.000000 \
#; -0.027147; 0.031415; -0.085335; 0.000000; 0.000000; 0.000000 \
#; 0.025013; -0.032280; 0.085946; 0.000000; 0.000000; 0.000000 \
#; 0.003782; -0.017444; -0.006074; 0.000000; 0.000000; 0.000000 \
#; 0.024920; -0.032621; 0.085976; 0.000000; 0.000000; 0.000000 \
#; 0.026296; -0.012527; -0.009561; 0.000000; 0.000000; 0.000000 \
#; -0.029566; 0.023259; 0.011138; 0.000000; 0.000000; 0.000000 \
```

```
#; 0.026378; -0.012648; -0.009445; 0.000000; 0.000000; 0.000000 \
#; -0.029416; 0.023076; 0.011079; 0.000000; 0.000000; 0.000000 \
# ]
#metaData: qpt=[0.000000;0.000000;0.000000;5.094454 \
#; -0.015600; -0.021658; 0.000043; 0.000000; 0.000000; 0.000000 \
#; 0.019839; 0.018813; 0.012653; 0.000000; 0.000000; 0.000000 \
#; 0.011542; 0.024625; -0.012872; 0.000000; 0.000000; 0.000000 \
#; -0.015532; -0.021457; 0.000014; 0.000000; 0.000000; 0.000000 \
#; -0.001290; -0.002255; -0.000168; 0.000000; 0.000000; 0.000000 \
#; -0.012062; -0.034617; 0.020867; 0.000000; 0.000000; 0.000000 \
#; -0.012771; -0.033561; 0.018302; 0.000000; 0.000000; 0.000000 \
#; -0.027977; -0.022906; -0.018606; 0.000000; 0.000000; 0.000000 \
#; -0.001604; -0.002390; -0.000037; 0.000000; 0.000000; 0.000000 \
#; -0.029089; -0.022570; -0.020776; 0.000000; 0.000000; 0.000000 \
#; -0.002237; 0.043677; -0.001440; 0.000000; 0.000000; 0.000000 \
#; 0.043307; 0.011150; 0.000875; 0.000000; 0.000000; 0.000000 \
#; -0.003455; 0.044805; -0.001150; 0.000000; 0.000000; 0.000000 \
#; 0.041947; 0.011949; 0.001183; 0.000000; 0.000000; 0.000000 \
# ]
#metaData: qpt=[0.000000;0.000000;0.000000;5.279731 \
#; -0.019053; 0.013963; 0.014016; 0.000000; 0.000000; 0.000000 \
#; 0.023164; -0.019119; -0.016042; 0.000000; 0.000000; 0.000000 \
#; 0.025609; -0.016075; -0.015726; 0.000000; 0.000000; 0.000000 \
#; -0.019416; 0.013955; 0.013938; 0.000000; 0.000000; 0.000000 \
#; -0.023626; 0.016842; 0.012980; 0.000000; 0.000000; 0.000000 \
#; -0.024003; 0.017180; 0.002265; 0.000000; 0.000000; 0.000000 \
#; -0.022527; 0.014336; 0.007757; 0.000000; 0.000000; 0.000000 \
#; -0.024287; 0.017492; 0.001193; 0.000000; 0.000000; 0.000000 \
#; -0.024257; 0.018003; 0.013778; 0.000000; 0.000000; 0.000000 \
#; -0.021848; 0.015992; 0.007723; 0.000000; 0.000000; 0.000000 \
```

```
#; -0.005647; 0.002306; 0.003894; 0.000000; 0.000000; 0.000000 \
#; -0.004123; 0.004154; 0.003824; 0.000000; 0.000000; 0.000000 \
#; -0.003855; 0.000765; 0.003461; 0.000000; 0.000000; 0.000000 \
#; -0.001386; 0.002713; 0.003079; 0.000000; 0.000000; 0.000000 \
# ]
#metaData: qpt=[0.000000;0.000000;0.000000;5.305514 \
#; -0.002620; -0.002353; 0.000410; 0.000000; 0.000000; 0.000000 \
#; 0.000451; -0.000778; -0.000711; 0.000000; 0.000000; 0.000000 \
#; 0.000664; -0.000739; -0.000198; 0.000000; 0.000000; 0.000000 \
#; 0.001809; 0.003699; 0.000294; 0.000000; 0.000000; 0.000000 \
#; 0.004307; 0.009294; 0.000783; 0.000000; 0.000000; 0.000000 \
#; 0.029999; -0.040372; 0.102379; 0.000000; 0.000000; 0.000000 \
#; -0.031348; 0.041834; -0.102539; 0.000000; 0.000000; 0.000000 \
#; 0.047631; -0.016330; 0.103414; 0.000000; 0.000000; 0.000000 \
#; -0.005629; -0.008073; 0.000049; 0.000000; 0.000000; 0.000000 \
#; -0.048018; 0.017094; -0.101310; 0.000000; 0.000000; 0.000000 \
#; 0.035509; -0.032128; -0.010951; 0.000000; 0.000000; 0.000000 \
#; 0.040612; -0.023241; -0.010005; 0.000000; 0.000000; 0.000000 \
#; -0.035362; 0.030905; 0.010786; 0.000000; 0.000000; 0.000000 \
#; -0.042252; 0.023554; 0.010604; 0.000000; 0.000000; 0.000000 \
# ]
#metaData: qpt=[0.000000;0.000000;0.000000;5.612879 \
#; 0.018097; -0.012892; 0.001961; 0.000000; 0.000000; 0.000000 \
#; -0.000106; 0.000019; 0.000173; 0.000000; 0.000000; 0.000000 \
#; -0.000102; 0.000060; 0.000023; 0.000000; 0.000000; 0.000000 \
#; -0.017952; 0.012873; -0.002062; 0.000000; 0.000000; 0.000000 \
#; 0.087644; -0.062659; -0.061163; 0.000000; 0.000000; 0.000000 \
#; -0.003252; -0.005796; 0.042486; 0.000000; 0.000000; 0.000000 \
#; 0.003101; 0.005730; -0.042991; 0.000000; 0.000000; 0.000000 \
#; -0.003766; -0.004988; -0.041398; 0.000000; 0.000000; 0.000000 \
```

```
#; -0.086598; 0.061774; 0.060673; 0.000000; 0.000000; 0.000000 \
#; 0.004177; 0.005017; 0.041518; 0.000000; 0.000000; 0.000000 \
#; -0.036555; 0.020654; 0.028394; 0.000000; 0.000000; 0.000000 \
#; 0.031100; -0.027711; -0.028076; 0.000000; 0.000000; 0.000000 \
#; 0.036650; -0.020771; -0.028698; 0.000000; 0.000000; 0.000000 \
#; -0.031618; 0.027883; 0.027706; 0.000000; 0.000000; 0.000000 \
# ]
#metaData: qpt=[0.000000;0.000000;0.000000;6.015065 \
#; 0.005087; 0.007042; -0.000132; 0.000000; 0.000000; 0.000000 \
#; 0.011952; -0.018420; 0.033970; 0.000000; 0.000000; 0.000000 \
#; -0.021311; 0.005054; -0.034120; 0.000000; 0.000000; 0.000000 \
#; 0.005064; 0.006941; -0.000168; 0.000000; 0.000000; 0.000000 \
#; -0.016300; -0.018482; 0.001223; 0.000000; 0.000000; 0.000000 \
#; 0.018802; 0.013899; 0.029206; 0.000000; 0.000000; 0.000000 \
#; 0.018016; 0.013968; 0.027846; 0.000000; 0.000000; 0.000000 \
#; 0.006904; 0.019902; -0.026644; 0.000000; 0.000000; 0.000000 \
#; -0.015757; -0.018650; 0.000955; 0.000000; 0.000000; 0.000000 \
#; 0.006748; 0.020438; -0.027831; 0.000000; 0.000000; 0.000000 \
#; -0.004028; -0.011585; 0.035023; 0.000000; 0.000000; 0.000000 \
#; -0.011008; -0.006965; -0.034053; 0.000000; 0.000000; 0.000000 \
#; -0.003420; -0.012101; 0.034670; 0.000000; 0.000000; 0.000000 \
#; -0.010150; -0.007461; -0.034453; 0.000000; 0.000000; 0.000000 \
# ]
#metaData: qpt=[0.000000;0.000000;0.000000;6.280402 \
#; -0.007596; -0.010436; 0.000093; 0.000000; 0.000000; 0.000000 \
#; 0.000053; -0.000180; 0.000165; 0.000000; 0.000000; 0.000000 \
#; -0.000195; 0.000042; -0.000167; 0.000000; 0.000000; 0.000000 \
#; 0.007675; 0.010634; 0.000043; 0.000000; 0.000000; 0.000000 \
#; 0.007399; 0.006213; -0.001122; 0.000000; 0.000000; 0.000000 \
#; -0.046647; -0.029050; -0.047416; 0.000000; 0.000000; 0.000000 \
```

```
#; 0.046131; 0.029050; 0.047071; 0.000000; 0.000000; 0.000000 \
#; 0.013136; 0.054011; -0.046302; 0.000000; 0.000000; 0.000000 \
#; -0.006117; -0.007515; 0.000227; 0.000000; 0.000000; 0.000000 \
#; -0.012816; -0.053080; 0.045348; 0.000000; 0.000000; 0.000000 \
#; 0.069712; -0.057099; -0.032800; 0.000000; 0.000000; 0.000000 \
#; 0.076021; -0.047390; -0.032762; 0.000000; 0.000000; 0.000000 \
#; -0.069541; 0.056520; 0.033311; 0.000000; 0.000000; 0.000000 \
#; -0.076408; 0.047369; 0.032438; 0.000000; 0.000000; 0.000000 \
# ]
#metaData: qpt=[0.000000;0.000000;0.000000;6.724682 \
#; -0.005889; 0.004981; 0.006491; 0.000000; 0.000000; 0.000000 \
#; -0.001841; -0.001185; -0.000369; 0.000000; 0.000000; 0.000000 \
#; -0.000506; 0.002275; -0.001213; 0.000000; 0.000000; 0.000000 \
#; -0.005793; 0.004831; 0.006468; 0.000000; 0.000000; 0.000000 \
#; 0.094941; -0.068117; -0.058770; 0.000000; 0.000000; 0.000000 \
#; -0.054146; -0.052833; -0.015751; 0.000000; 0.000000; 0.000000 \
#; -0.054718; -0.052689; -0.016592; 0.000000; 0.000000; 0.000000 \
#; 0.029418; 0.065533; -0.017219; 0.000000; 0.000000; 0.000000 \
#; 0.095389; -0.068209; -0.058943; 0.000000; 0.000000; 0.000000 \
#; 0.029283; 0.065646; -0.018114; 0.000000; 0.000000; 0.000000 \
#; 0.011415; -0.018058; 0.006093; 0.000000; 0.000000; 0.000000 \
#; 0.020625; -0.006059; 0.003848; 0.000000; 0.000000; 0.000000 \
#; 0.012021; -0.018470; 0.005905; 0.000000; 0.000000; 0.000000 \
#; 0.022496; -0.007180; 0.002991; 0.000000; 0.000000; 0.000000 \
# ]
#metaData: qpt=[0.000000;0.000000;0.000000;7.495758 \
#; -0.012447; -0.012307; -0.002952; 0.000000; 0.000000; 0.000000 \
#; -0.000180; 0.002643; -0.001770; 0.000000; 0.000000; 0.000000 \
#; 0.003579; 0.000715; 0.000743; 0.000000; 0.000000; 0.000000 \
#; -0.012521; -0.012389; -0.003028; 0.000000; 0.000000; 0.000000 \
```

```
#; 0.003620; -0.005262; -0.002529; 0.000000; 0.000000; 0.000000 \
#; 0.072649; 0.070731; 0.040453; 0.000000; 0.000000; 0.000000 \
#; 0.072083; 0.070429; 0.040177; 0.000000; 0.000000; 0.000000 \
#; 0.068186; 0.087159; 0.016331; 0.000000; 0.000000; 0.000000 \
#; 0.003677; -0.006381; -0.002817; 0.000000; 0.000000; 0.000000 \
#; 0.068466; 0.087236; 0.016967; 0.000000; 0.000000; 0.000000 \
#; 0.023532; -0.009628; -0.022410; 0.000000; 0.000000; 0.000000 \
#; -0.007486; 0.015415; 0.021299; 0.000000; 0.000000; 0.000000 \
#; 0.023557; -0.009492; -0.023107; 0.000000; 0.000000; 0.000000 \
#; -0.008387; 0.015979; 0.021241; 0.000000; 0.000000; 0.000000 \
# ]
#metaData: qpt=[0.000000;0.000000;0.000000;7.542987 \
#; 0.004998; -0.009410; 0.011340; 0.000000; 0.000000; 0.000000 \
#; -0.000537; 0.002471; 0.001345; 0.000000; 0.000000; 0.000000 \
#; -0.001022; 0.000063; 0.001967; 0.000000; 0.000000; 0.000000 \
#; 0.004895; -0.009506; 0.011444; 0.000000; 0.000000; 0.000000 \
#; -0.012229; 0.008236; 0.005524; 0.000000; 0.000000; 0.000000 \
#; -0.007714; 0.059922; -0.098587; 0.000000; 0.000000; 0.000000 \
#; -0.008640; 0.058939; -0.098998; 0.000000; 0.000000; 0.000000 \
#; -0.027288; 0.038913; -0.105692; 0.000000; 0.000000; 0.000000 \
#; -0.012152; 0.007712; 0.005314; 0.000000; 0.000000; 0.000000 \
#; -0.027691; 0.039471; -0.106858; 0.000000; 0.000000; 0.000000 \
#; -0.001395; 0.007040; -0.004081; 0.000000; 0.000000; 0.000000 \
#; -0.012416; 0.007834; 0.007467; 0.000000; 0.000000; 0.000000 \
#; -0.001066; 0.006713; -0.004236; 0.000000; 0.000000; 0.000000 \
#; -0.013542; 0.008393; 0.007433; 0.000000; 0.000000; 0.000000 \
# ]
#metaData: qpt=[0.000000;0.000000;0.000000;7.990594 \
#; 0.006097; -0.004717; -0.004244; 0.000000; 0.000000; 0.000000 \
#; 0.001880; -0.006884; -0.001418; 0.000000; 0.000000; 0.000000 \
```

```
#; 0.006913; 0.000535; -0.001160; 0.000000; 0.000000; 0.000000 \
#; 0.006086; -0.004510; -0.004693; 0.000000; 0.000000; 0.000000 \
#; 0.000442; 0.002454; 0.000414; 0.000000; 0.000000; 0.000000 \
#; -0.015017; -0.038523; 0.004592; 0.000000; 0.000000; 0.000000 \
#; -0.015056; -0.034384; 0.000422; 0.000000; 0.000000; 0.000000 \
#; 0.030941; 0.029525; 0.001277; 0.000000; 0.000000; 0.000000 \
#; 0.000105; -0.002788; -0.000041; 0.000000; 0.000000; 0.000000 \
#; 0.031801; 0.026998; 0.006162; 0.000000; 0.000000; 0.000000 \
#; -0.082940; 0.067256; 0.042110; 0.000000; 0.000000; 0.000000 \
#; -0.090955; 0.056396; 0.041566; 0.000000; 0.000000; 0.000000 \
#; -0.083427; 0.070848; 0.035854; 0.000000; 0.000000; 0.000000 \
#; -0.089260; 0.054714; 0.036294; 0.000000; 0.000000; 0.000000 \
# ]
#metaData: qpt=[0.000000;0.000000;0.000000;8.029669 \
#; 0.000735; 0.000674; -0.000088; 0.000000; 0.000000; 0.000000 \
#; 0.000065; -0.000249; -0.000053; 0.000000; 0.000000; 0.000000 \
#; 0.000148; 0.000019; -0.000025; 0.000000; 0.000000; 0.000000 \
#; -0.000450; -0.001001; -0.000171; 0.000000; 0.000000; 0.000000 \
#; -0.047772; -0.068549; -0.001212; 0.000000; 0.000000; 0.000000 \
#; 0.013698; 0.024228; 0.004401; 0.000000; 0.000000; 0.000000 \
#; -0.014219; -0.025562; -0.004966; 0.000000; 0.000000; 0.000000 \
#; -0.017128; -0.021618; 0.007283; 0.000000; 0.000000; 0.000000 \
#; 0.047745; 0.068281; 0.001156; 0.000000; 0.000000; 0.000000 \
#; 0.019834; 0.024051; -0.006177; 0.000000; 0.000000; 0.000000 \
#; 0.003389; 0.033846; -0.103467; 0.000000; 0.000000; 0.000000 \
#; -0.025414; -0.016085; -0.097292; 0.000000; 0.000000; 0.000000 \
#; -0.009345; -0.028771; 0.105875; 0.000000; 0.000000; 0.000000 \
#; 0.021744; 0.018496; 0.099356; 0.000000; 0.000000; 0.000000 \
# ]
#metaData: qpt=[0.000000;0.000000;0.000000;8.066696 \
```

```
#; 0.003193; -0.001524; 0.009339; 0.000000; 0.000000; 0.000000 \
#; -0.000112; 0.000288; 0.000072; 0.000000; 0.000000; 0.000000 \
#; -0.000093; -0.000023; 0.000032; 0.000000; 0.000000; 0.000000 \
#; -0.003363; 0.001717; -0.009132; 0.000000; 0.000000; 0.000000 \
#; -0.050874; 0.036535; 0.013021; 0.000000; 0.000000; 0.000000 \
#; 0.035219; -0.029436; 0.091798; 0.000000; 0.000000; 0.000000 \
#; -0.035052; 0.030632; -0.092066; 0.000000; 0.000000; 0.000000 \
#; -0.046365; 0.016297; -0.094718; 0.000000; 0.000000; 0.000000 \
#; 0.050647; -0.036069; -0.012880; 0.000000; 0.000000; 0.000000 \
#; 0.044007; -0.018421; 0.093436; 0.000000; 0.000000; 0.000000 \
#; 0.020658; -0.043114; 0.010595; 0.000000; 0.000000; 0.000000 \
#; -0.038294; -0.000582; -0.016393; 0.000000; 0.000000; 0.000000 \
#; -0.015136; 0.038264; -0.012610; 0.000000; 0.000000; 0.000000 \
#; 0.040816; -0.001017; 0.015166; 0.000000; 0.000000; 0.000000 \
# ]
#metaData: qpt=[0.000000;0.000000;0.000000;8.262569 \
#; -0.007786; -0.011081; 0.000293; 0.000000; 0.000000; 0.000000 \
#; -0.000193; 0.000788; 0.000166; 0.000000; 0.000000; 0.000000 \
#; 0.000746; 0.000057; -0.000134; 0.000000; 0.000000; 0.000000 \
#; 0.007594; 0.010771; -0.000213; 0.000000; 0.000000; 0.000000 \
#; -0.022902; -0.030016; 0.000041; 0.000000; 0.000000; 0.000000 \
#; -0.054588; -0.084404; 0.018572; 0.000000; 0.000000; 0.000000 \
#; 0.054765; 0.080857; -0.011397; 0.000000; 0.000000; 0.000000 \
#; 0.052563; 0.076244; 0.004390; 0.000000; 0.000000; 0.000000 \
#; 0.024294; 0.032758; 0.000195; 0.000000; 0.000000; 0.000000 \
#; -0.057571; -0.078669; -0.012689; 0.000000; 0.000000; 0.000000 \
#; -0.034582; 0.035020; -0.002711; 0.000000; 0.000000; 0.000000 \
#; -0.067060; 0.031493; -0.002448; 0.000000; 0.000000; 0.000000 \
#; 0.047128; -0.047025; 0.000769; 0.000000; 0.000000; 0.000000 \
#; 0.052637; -0.024233; 0.003583; 0.000000; 0.000000; 0.000000 \
```

```
# ]
#metaData: qpt=[0.000000;0.000000;0.000000;8.333743 \
#; -0.000073; 0.000041; 0.000111; 0.000000; 0.000000; 0.000000 \
#; 0.002706; -0.009164; -0.002580; 0.000000; 0.000000; 0.000000 \
#; -0.009671; -0.000340; 0.002635; 0.000000; 0.000000; 0.000000 \
#; 0.001129; 0.001849; -0.000072; 0.000000; 0.000000; 0.000000 \
#; -0.014445; -0.018980; 0.000156; 0.000000; 0.000000; 0.000000 \
#; 0.001149; 0.026528; -0.046235; 0.000000; 0.000000; 0.000000 \
#; 0.009157; 0.040332; -0.050453; 0.000000; 0.000000; 0.000000 \
#; 0.033418; 0.021548; 0.048703; 0.000000; 0.000000; 0.000000 \
#; -0.010141; -0.015580; -0.000222; 0.000000; 0.000000; 0.000000 \
#; 0.025044; 0.008364; 0.049098; 0.000000; 0.000000; 0.000000 \
#; -0.076699; 0.075364; 0.008515; 0.000000; 0.000000; 0.000000 \
#; 0.090416; -0.045604; -0.008523; 0.000000; 0.000000; 0.000000 \
#; -0.070448; 0.070272; 0.006101; 0.000000; 0.000000; 0.000000 \
#; 0.099758; -0.049860; -0.008887; 0.000000; 0.000000; 0.000000 \
# ]
#metaData: qpt=[0.000000;0.000000;0.000000;8.732081 \
#; -0.000046; -0.000351; 0.004235; 0.000000; 0.000000; 0.000000 \
#; 0.000023; -0.000031; -0.000001; 0.000000; 0.000000; 0.000000 \
#; 0.000071; 0.000007; 0.000008; 0.000000; 0.000000; 0.000000 \
#; 0.000061; 0.000456; -0.004296; 0.000000; 0.000000; 0.000000 \
#; -0.046504; 0.032756; 0.021319; 0.000000; 0.000000; 0.000000 \
#; 0.048080; 0.052173; 0.015661; 0.000000; 0.000000; 0.000000 \
#; -0.048810; -0.053404; -0.015065; 0.000000; 0.000000; 0.000000 \
#; 0.037524; 0.067537; -0.014616; 0.000000; 0.000000; 0.000000 \
#; 0.046842; -0.032816; -0.021650; 0.000000; 0.000000; 0.000000 \
#; -0.037587; -0.068025; 0.015268; 0.000000; 0.000000; 0.000000 \
#; -0.066203; 0.060612; -0.005257; 0.000000; 0.000000; 0.000000 \
#; 0.076136; -0.041340; 0.006194; 0.000000; 0.000000; 0.000000 \
```

```
#; 0.065790; -0.060354; 0.005358; 0.000000; 0.000000; 0.000000 \
#; -0.076969; 0.041691; -0.006452; 0.000000; 0.000000; 0.000000 \
# ]
#metaData: qpt=[0.000000;0.000000;0.000000;8.790231 \
#; -0.001792; 0.001261; -0.005988; 0.000000; 0.000000; 0.000000 \
#; 0.000009; -0.000048; 0.000009; 0.000000; 0.000000; 0.000000 \
#; 0.000101; 0.000028; 0.000014; 0.000000; 0.000000; 0.000000 \
#; 0.001757; -0.001230; 0.006030; 0.000000; 0.000000; 0.000000 \
#; -0.004797; 0.000390; 0.034946; 0.000000; 0.000000; 0.000000 \
#; 0.012222; 0.025660; -0.018500; 0.000000; 0.000000; 0.000000 \
#; -0.012559; -0.026286; 0.018885; 0.000000; 0.000000; 0.000000 \
#; 0.019593; 0.018935; 0.019521; 0.000000; 0.000000; 0.000000 \
#; 0.004998; -0.000194; -0.035079; 0.000000; 0.000000; 0.000000 \
#; -0.019574; -0.018981; -0.019664; 0.000000; 0.000000; 0.000000 \
#; 0.004450; -0.017534; 0.112117; 0.000000; 0.000000; 0.000000 \
#; -0.019145; -0.002410; -0.117842; 0.000000; 0.000000; 0.000000 \
#; -0.004485; 0.017562; -0.112452; 0.000000; 0.000000; 0.000000 \
#; 0.018187; 0.002726; 0.117113; 0.000000; 0.000000; 0.000000 \
# ]
#metaData: qpt=[0.000000;0.000000;0.000000;9.255582 \
#; -0.000596; 0.000087; -0.005760; 0.000000; 0.000000; 0.000000 \
#; 0.005592; 0.013148; -0.008717; 0.000000; 0.000000; 0.000000 \
#; -0.010481; -0.009240; -0.008156; 0.000000; 0.000000; 0.000000 \
#; -0.000605; 0.000116; -0.005730; 0.000000; 0.000000; 0.000000 \
#; -0.004186; 0.005775; 0.004652; 0.000000; 0.000000; 0.000000 \
#; 0.003770; 0.006977; -0.005598; 0.000000; 0.000000; 0.000000 \
#; 0.003798; 0.006929; -0.005507; 0.000000; 0.000000; 0.000000 \
#; -0.004323; -0.005882; -0.004201; 0.000000; 0.000000; 0.000000 \
#; -0.004378; 0.005995; 0.004584; 0.000000; 0.000000; 0.000000 \
#; -0.004569; -0.006055; -0.004359; 0.000000; 0.000000; 0.000000 \
```

```
#; 0.009586; -0.039691; 0.110241; 0.000000; 0.000000; 0.000000 \
#; 0.040443; 0.002409; 0.105056; 0.000000; 0.000000; 0.000000 \
#; 0.010019; -0.040102; 0.109415; 0.000000; 0.000000; 0.000000 \
#; 0.039982; 0.002689; 0.105921; 0.000000; 0.000000; 0.000000 \
# ]
#metaData: qpt=[0.000000;0.000000;0.000000;9.799399 \
#; 0.005274; 0.007385; -0.000105; 0.000000; 0.000000; 0.000000 \
#; -0.003551; -0.001920; 0.004239; 0.000000; 0.000000; 0.000000 \
#; -0.001118; -0.004416; -0.004622; 0.000000; 0.000000; 0.000000 \
#; 0.005265; 0.007352; -0.000090; 0.000000; 0.000000; 0.000000 \
#; -0.062565; -0.087650; 0.000048; 0.000000; 0.000000; 0.000000 \
#; 0.004158; -0.016745; 0.017618; 0.000000; 0.000000; 0.000000 \
#; 0.004050; -0.016950; 0.017896; 0.000000; 0.000000; 0.000000 \
#; -0.017474; -0.001705; -0.018236; 0.000000; 0.000000; 0.000000 \
#; -0.062770; -0.087811; -0.000025; 0.000000; 0.000000; 0.000000 \
#; -0.017670; -0.001642; -0.018350; 0.000000; 0.000000; 0.000000 \
#; 0.012672; 0.024471; -0.083801; 0.000000; 0.000000; 0.000000 \
#; 0.020564; 0.020482; 0.089028; 0.000000; 0.000000; 0.000000 \
#; 0.012740; 0.024300; -0.083709; 0.000000; 0.000000; 0.000000 \
#; 0.020511; 0.020540; 0.089523; 0.000000; 0.000000; 0.000000 \
# ]
#metaData: qpt=[0.000000;0.000000;0.000000;11.900520 \
#; -0.001431; -0.002000; -0.000060; 0.000000; 0.000000; 0.000000 \
#; -0.000002; 0.000052; -0.000024; 0.000000; 0.000000; 0.000000 \
#; 0.000055; 0.000015; 0.000023; 0.000000; 0.000000; 0.000000 \
#; 0.001414; 0.002000; 0.000066; 0.000000; 0.000000; 0.000000 \
#; -0.085552; -0.119176; -0.000393; 0.000000; 0.000000; 0.000000 \
#; -0.007764; 0.018345; 0.007049; 0.000000; 0.000000; 0.000000 \
#; 0.007652; -0.018222; -0.006988; 0.000000; 0.000000; 0.000000 \
#; -0.019722; 0.001137; 0.007003; 0.000000; 0.000000; 0.000000 \
```

```
#; 0.084967; 0.118369; 0.000386; 0.000000; 0.000000; 0.000000 \
#; 0.020104; -0.001235; -0.007152; 0.000000; 0.000000; 0.000000 \
#; 0.021626; -0.024633; 0.057842; 0.000000; 0.000000; 0.000000 \
#; 0.029639; -0.012628; 0.058613; 0.000000; 0.000000; 0.000000 \
#; -0.021303; 0.024227; -0.057806; 0.000000; 0.000000; 0.000000 \
#; -0.030176; 0.012835; -0.058628; 0.000000; 0.000000; 0.000000 \
# ]
#metaData: qpt=[0.000000;0.000000;0.000000;12.255137 \
#; 0.000604; 0.000829; -0.000054; 0.000000; 0.000000; 0.000000 \
#; -0.004348; -0.011280; 0.011244; 0.000000; 0.000000; 0.000000 \
#; -0.009344; -0.007770; -0.011254; 0.000000; 0.000000; 0.000000 \
#; 0.000647; 0.000850; -0.000052; 0.000000; 0.000000; 0.000000 \
#; 0.074371; 0.103749; 0.000072; 0.000000; 0.000000; 0.000000 \
#; 0.023366; -0.024340; -0.008103; 0.000000; 0.000000; 0.000000 \
#; 0.023656; -0.024450; -0.008180; 0.000000; 0.000000; 0.000000 \
#; -0.031038; 0.014264; 0.008300; 0.000000; 0.000000; 0.000000 \
#; 0.075038; 0.104494; 0.000254; 0.000000; 0.000000; 0.000000 \
#; -0.031206; 0.014181; 0.008334; 0.000000; 0.000000; 0.000000 \
#; 0.001926; 0.024175; -0.059121; 0.000000; 0.000000; 0.000000 \
#; 0.022649; 0.009597; 0.060065; 0.000000; 0.000000; 0.000000 \
#; 0.001901; 0.024344; -0.059521; 0.000000; 0.000000; 0.000000 \
#; 0.022537; 0.009752; 0.059805; 0.000000; 0.000000; 0.000000 \
# ]
#metaData: qpt=[0.000000;0.000000;0.000000;13.318135 \
#; -0.003487; 0.002502; -0.006938; 0.000000; 0.000000; 0.000000 \
#; 0.000016; 0.000022; -0.000005; 0.000000; 0.000000; 0.000000 \
#; -0.000001; -0.000008; 0.000008; 0.000000; 0.000000; 0.000000 \
#; 0.003494; -0.002510; 0.006918; 0.000000; 0.000000; 0.000000 \
#; -0.035995; 0.026312; -0.097412; 0.000000; 0.000000; 0.000000 \
#; -0.007797; 0.011179; -0.013411; 0.000000; 0.000000; 0.000000 \
```

```
#; 0.007787; -0.011216; 0.013579; 0.000000; 0.000000; 0.000000 \
#; 0.013056; -0.003733; 0.013620; 0.000000; 0.000000; 0.000000 \
#; 0.035848; -0.026455; 0.097504; 0.000000; 0.000000; 0.000000 \
#; -0.013098; 0.003785; -0.013593; 0.000000; 0.000000; 0.000000 \
#; -0.064128; -0.070185; -0.001885; 0.000000; 0.000000; 0.000000 \
#; -0.045776; -0.083105; 0.001810; 0.000000; 0.000000; 0.000000 \
#; 0.063960; 0.069894; 0.001952; 0.000000; 0.000000; 0.000000 \
#; 0.045822; 0.083408; -0.001940; 0.000000; 0.000000; 0.000000 \
# ]
#metaData: qpt=[0.000000;0.000000;0.000000;15.661149 \
#; 0.000031; 0.000083; 0.000004; 0.000000; 0.000000; 0.000000 \
#; -0.000065; -0.000064; -0.000012; 0.000000; 0.000000; 0.000000 \
#; -0.000055; -0.000100; 0.000022; 0.000000; 0.000000; 0.000000 \
#; 0.000051; -0.000125; -0.000042; 0.000000; 0.000000; 0.000000 \
#; -0.003022; -0.004120; -0.000298; 0.000000; 0.000000; 0.000000 \
#; 0.057631; -0.041765; -0.033055; 0.000000; 0.000000; 0.000000 \
#; -0.058985; 0.042568; 0.033777; 0.000000; 0.000000; 0.000000 \
#; 0.059961; -0.041970; -0.033986; 0.000000; 0.000000; 0.000000 \
#; 0.003203; 0.004272; 0.000212; 0.000000; 0.000000; 0.000000 \
#; -0.059892; 0.041725; 0.033891; 0.000000; 0.000000; 0.000000 \
#; 0.065334; 0.067777; 0.023060; 0.000000; 0.000000; 0.000000 \
#; -0.042210; -0.082030; 0.022375; 0.000000; 0.000000; 0.000000 \
#; -0.064375; -0.066868; -0.022708; 0.000000; 0.000000; 0.000000 \
#; 0.042919; 0.083466; -0.022864; 0.000000; 0.000000; 0.000000 \
# ]
#metaData: qpt=[0.000000;0.000000;0.000000;16.924279 \
#; -0.000599; 0.000436; -0.001175; 0.000000; 0.000000; 0.000000 \
#; -0.006965; -0.001766; -0.008964; 0.000000; 0.000000; 0.000000 \
#; -0.000307; 0.007648; -0.009002; 0.000000; 0.000000; 0.000000 \
#; -0.000573; 0.000419; -0.001184; 0.000000; 0.000000; 0.000000 \
```

#; 0.055119; -0.039962; 0.139807; 0.000000; 0.000000; 0.000000 \
#; -0.001088; -0.000510; -0.002845; 0.000000; 0.000000; 0.000000 \
#; -0.001129; -0.000523; -0.002875; 0.000000; 0.000000; 0.000000 \
#; -0.001683; 0.002661; -0.001815; 0.000000; 0.000000; 0.000000 \
#; 0.055381; -0.040124; 0.139983; 0.000000; 0.000000; 0.000000 \
#; -0.001852; 0.002754; -0.001720; 0.000000; 0.000000; 0.000000 \
#; 0.032979; 0.033885; 0.007200; 0.000000; 0.000000; 0.000000 \
#; -0.023139; -0.045649; 0.007922; 0.000000; 0.000000; 0.000000 \
#; 0.032949; 0.033739; 0.007070; 0.000000; 0.000000; 0.000000 \
#; -0.023187; -0.045577; 0.008004; 0.000000; 0.000000; 0.000000 \
]
#metaData: qpt=[0.000000;0.000000;0.000000;17.107277 \
#; 0.000119; 0.000065; 0.000009; 0.000000; 0.000000; 0.000000 \
#; 0.009339; 0.009663; 0.001872; 0.000000; 0.000000; 0.000000 \
#; 0.006156; 0.011688; -0.001486; 0.000000; 0.000000; 0.000000 \
#; 0.000196; 0.000020; -0.000035; 0.000000; 0.000000; 0.000000 \
#; -0.008924; -0.010019; -0.003195; 0.000000; 0.000000; 0.000000 \
#; 0.050623; -0.033259; -0.028947; 0.000000; 0.000000; 0.000000 \
#; 0.050407; -0.033064; -0.028845; 0.000000; 0.000000; 0.000000 \
#; -0.048266; 0.037028; 0.029053; 0.000000; 0.000000; 0.000000 \
#; -0.008721; -0.010166; -0.002726; 0.000000; 0.000000; 0.000000 \
#; -0.049669; 0.038010; 0.029904; 0.000000; 0.000000; 0.000000 \
#; -0.066830; -0.068099; -0.022303; 0.000000; 0.000000; 0.000000 \
#; -0.042679; -0.084986; 0.022385; 0.000000; 0.000000; 0.000000 \
#; -0.068178; -0.069379; -0.022851; 0.000000; 0.000000; 0.000000 \
#; -0.042207; -0.083988; 0.022145; 0.000000; 0.000000; 0.000000 \
]
#metaData: qpt=[0.000000;0.000000;0.000000;18.081925 \
#; -0.000661; 0.000469; -0.000832; 0.000000; 0.000000; 0.000000 \
#; -0.007371; -0.010357; 0.001214; 0.000000; 0.000000; 0.000000 \

```
#; 0.007404; 0.010301; 0.001229; 0.000000; 0.000000; 0.000000 \
#; -0.000602; 0.000427; -0.000857; 0.000000; 0.000000; 0.000000 \
#; -0.026463; 0.018969; -0.067269; 0.000000; 0.000000; 0.000000 \
#; 0.000439; 0.000769; -0.000089; 0.000000; 0.000000; 0.000000 \
#; 0.000724; 0.000543; -0.000261; 0.000000; 0.000000; 0.000000 \
#; -0.000228; -0.000941; -0.000474; 0.000000; 0.000000; 0.000000 \
#; -0.026233; 0.018817; -0.066415; 0.000000; 0.000000; 0.000000 \
#; -0.000707; -0.000633; -0.000219; 0.000000; 0.000000; 0.000000 \
#; 0.076621; 0.069779; 0.030976; 0.000000; 0.000000; 0.000000 \
#; -0.041157; -0.094686; 0.030947; 0.000000; 0.000000; 0.000000 \
#; 0.076464; 0.069634; 0.030829; 0.000000; 0.000000; 0.000000 \
#; -0.041154; -0.094761; 0.031045; 0.000000; 0.000000; 0.000000 \
# ]
#metaData: qpt=[0.000000;0.000000;0.000000;19.848633 \
#; 0.007685; -0.005460; -0.003674; 0.000000; 0.000000; 0.000000 \
#; 0.000047; 0.000056; 0.000063; 0.000000; 0.000000; 0.000000 \
#; 0.000088; 0.000137; -0.000072; 0.000000; 0.000000; 0.000000 \
#; -0.007659; 0.005452; 0.003643; 0.000000; 0.000000; 0.000000 \
#; 0.037841; -0.026288; 0.092435; 0.000000; 0.000000; 0.000000 \
#; 0.050724; -0.036889; -0.030522; 0.000000; 0.000000; 0.000000 \
#; -0.052722; 0.038366; 0.031687; 0.000000; 0.000000; 0.000000 \
#; -0.051754; 0.035916; 0.030698; 0.000000; 0.000000; 0.000000 \
#; -0.037373; 0.027160; -0.092597; 0.000000; 0.000000; 0.000000 \
#; 0.053412; -0.037030; -0.031616; 0.000000; 0.000000; 0.000000 \
#; -0.045240; -0.044417; -0.021445; 0.000000; 0.000000; 0.000000 \
#; -0.028476; -0.059331; 0.022107; 0.000000; 0.000000; 0.000000 \
#; 0.043688; 0.043544; 0.020198; 0.000000; 0.000000; 0.000000 \
#; 0.027524; 0.056255; -0.020351; 0.000000; 0.000000; 0.000000 \
# ]
#metaData: qpt=[0.000000;0.000000;0.000000;20.679948 \
```

#; -0.000363; 0.000550; 0.000270; 0.000000; 0.000000; 0.000000 \
#; 0.002852; 0.004081; 0.003634; 0.000000; 0.000000; 0.000000 \
#; 0.002954; 0.004022; -0.003642; 0.000000; 0.000000; 0.000000 \
#; -0.000176; 0.000416; 0.000202; 0.000000; 0.000000; 0.000000 \
#; 0.016479; 0.024864; -0.001616; 0.000000; 0.000000; 0.000000 \
#; -0.069855; 0.050645; 0.039818; 0.000000; 0.000000; 0.000000 \
#; -0.069245; 0.050190; 0.039487; 0.000000; 0.000000; 0.000000 \
#; 0.077491; -0.054219; -0.043627; 0.000000; 0.000000; 0.000000 \
#; 0.017919; 0.023987; 0.001837; 0.000000; 0.000000; 0.000000 \
#; 0.075876; -0.053127; -0.042686; 0.000000; 0.000000; 0.000000 \
#; -0.050168; -0.029603; -0.039786; 0.000000; 0.000000; 0.000000 \
#; -0.011771; -0.056488; 0.039941; 0.000000; 0.000000; 0.000000 \
#; -0.051368; -0.030905; -0.040387; 0.000000; 0.000000; 0.000000 \
#; -0.012843; -0.058906; 0.040710; 0.000000; 0.000000; 0.000000 \
]
#metaData: qpt=[0.000000;0.000000;0.000000;21.636555 \
#; 0.011409; -0.008121; -0.006386; 0.000000; 0.000000; 0.000000 \
#; 0.000103; 0.000309; 0.000218; 0.000000; 0.000000; 0.000000 \
#; -0.000103; -0.000013; 0.000003; 0.000000; 0.000000; 0.000000 \
#; 0.011438; -0.008109; -0.006389; 0.000000; 0.000000; 0.000000 \
#; 0.003635; -0.002299; -0.003283; 0.000000; 0.000000; 0.000000 \
#; -0.089259; 0.062572; 0.049614; 0.000000; 0.000000; 0.000000 \
#; -0.091918; 0.064487; 0.051184; 0.000000; 0.000000; 0.000000 \
#; -0.082397; 0.059449; 0.046233; 0.000000; 0.000000; 0.000000 \
#; 0.004604; -0.001172; -0.002952; 0.000000; 0.000000; 0.000000 \
#; -0.085194; 0.061528; 0.047857; 0.000000; 0.000000; 0.000000 \
#; -0.001107; -0.002984; -0.002076; 0.000000; 0.000000; 0.000000 \
#; 0.002032; -0.000830; 0.000119; 0.000000; 0.000000; 0.000000 \
#; 0.001459; -0.000085; -0.001528; 0.000000; 0.000000; 0.000000 \
#; -0.000046; -0.004779; 0.000948; 0.000000; 0.000000; 0.000000 \

]
#metaData: qpt=[0.000000;0.000000;0.000000;22.457750 \
#; -0.000612; -0.000175; 0.000211; 0.000000; 0.000000; 0.000000 \
#; -0.000001; -0.000016; -0.000016; 0.000000; 0.000000; 0.000000 \
#; -0.000008; -0.000007; 0.000012; 0.000000; 0.000000; 0.000000 \
#; 0.000091; 0.000539; 0.000080; 0.000000; 0.000000; 0.000000 \
#; -0.016845; -0.024766; 0.001431; 0.000000; 0.000000; 0.000000 \
#; 0.071323; -0.050016; -0.040831; 0.000000; 0.000000; 0.000000 \
#; -0.066700; 0.046836; 0.038284; 0.000000; 0.000000; 0.000000 \
#; 0.071397; -0.051605; -0.041339; 0.000000; 0.000000; 0.000000 \
#; 0.016557; 0.024701; -0.001316; 0.000000; 0.000000; 0.000000 \
#; -0.068098; 0.049263; 0.039478; 0.000000; 0.000000; 0.000000 \
#; -0.053472; -0.058562; -0.012822; 0.000000; 0.000000; 0.000000 \
#; 0.037229; 0.068081; -0.012615; 0.000000; 0.000000; 0.000000 \
#; 0.053674; 0.058699; 0.013152; 0.000000; 0.000000; 0.000000 \
#; -0.037230; -0.067674; 0.012302; 0.000000; 0.000000; 0.000000 \
]
#metaData: qpt=[0.000000;0.000000;0.000000;24.756879 \
#; 0.007489; -0.005330; -0.005818; 0.000000; 0.000000; 0.000000 \
#; -0.000006; -0.000005; -0.000004; 0.000000; 0.000000; 0.000000 \
#; -0.000005; -0.000001; 0.000009; 0.000000; 0.000000; 0.000000 \
#; -0.007506; 0.005341; 0.005829; 0.000000; 0.000000; 0.000000 \
#; -0.029802; 0.020846; -0.079520; 0.000000; 0.000000; 0.000000 \
#; 0.071663; -0.048990; -0.042022; 0.000000; 0.000000; 0.000000 \
#; -0.070782; 0.048446; 0.041582; 0.000000; 0.000000; 0.000000 \
#; -0.067420; 0.049850; 0.040676; 0.000000; 0.000000; 0.000000 \
#; 0.029704; -0.020964; 0.079436; 0.000000; 0.000000; 0.000000 \
#; 0.066828; -0.049513; -0.040342; 0.000000; 0.000000; 0.000000 \
#; 0.030657; 0.030129; 0.010536; 0.000000; 0.000000; 0.000000 \
#; 0.019372; 0.040167; -0.010896; 0.000000; 0.000000; 0.000000 \

```
#; -0.030443; -0.030024; -0.010376; 0.000000; 0.000000; 0.000000 \
#; -0.019374; -0.040010; 0.010730; 0.000000; 0.000000; 0.000000 \
# ]
```

References and Notes

1. Tori Z Forbes, Peter C Burns, S Skanthakumar, and L Soderholm. Synthesis, structure, and magnetism of Np_2O_5. *Journal of the American Chemical Society*, 129(10):2760–2761, 2007.

2. Y Yun, Jan Rusz, M-T Suzuki, and PM Oppeneer. First-principles investigation of higher oxides of uranium and neptunium: U_3O_8 and Np_2O_5. *Physical Review B*, 83(7):075109, 2011.
